# Coordinated regulation of gene expression and microRNA changes in adipose tissue and circulating extracellular vesicles in response to pioglitazone treatment in humans with type 2 diabetes

**DOI:** 10.3389/fendo.2022.955593

**Published:** 2022-08-31

**Authors:** Yury O. Nunez Lopez, Anna Casu, Zuzana Kovacova, Alejandra M. Petrilli, Olga Sideleva, William G. Tharp, Richard E. Pratley

**Affiliations:** ^1^ Diabetes Program, Translational Research Institute, AdventHealth, Orlando, FL, United States; ^2^ Department of Medicine, Larner College of Medicine, University of Vermont, Burlington, VT, United States; ^3^ Department of Anesthesiology, University of Vermont Medical Center, University of Vermont Larner College of Medicine, Burlington, VT, United States

**Keywords:** type 2 diabetes, pioglitazone, adipose tissue, exosome, extracellular vesicle, microRNA, gene expression, circulating EV

## Abstract

Pioglitazone, a PPARγ agonist, is used to treat type 2 diabetes (T2D). PPARγ is highly expressed in adipose tissue (AT), however the effects of pioglitazone to improve insulin sensitivity are also evident in other tissues and PPARγ agonism has been shown to alter cancer derived extracellular vesicle (EV)-miRNAs. We hypothesized that pioglitazone modifies the cargo of circulating AT-derived EVs to alter interorgan crosstalk in people with diabetes. We tested our hypothesis in a 3-month trial in which 24 subjects with T2D were randomized to treatment with either pioglitazone 45 mg/day or placebo (NCT00656864). Levels of 42 adipocyte-derived EV-miRNAs were measured in plasma EVs using low density TaqMan arrays. Levels of differentially expressed EV-miRNAs and their most relevant target genes were also measure in adipose tissue from the same participants, using individual TaqMan assays. Levels of 5 miRNAs (i.e., miR-7-5p, miR-20a-5p, miR-92a-3p, miR-195-5p, and miR-374b-5p) were significantly downregulated in EVs in response to pioglitazone treatment relative to placebo. The opposite occurred for miR-195-5p in subcutaneous AT. Changes in miRNA expression in EVs and AT correlated with changes in suppression of lipolysis and improved insulin sensitivity, among others. DICER was downregulated and exosomal miRNA sorting-related genes YBX1 and hnRNPA2B1 displayed a downregulation trend in AT. Furthermore, analysis of EV-miRNA targeted genes identified a network of transcripts that changed in a coordinated manner in AT. Collectively, our results suggest that some beneficial pharmacologic effects of pioglitazone are mediated by adipose-specific miRNA regulation and exosomal/EV trafficking.

**Clinical Trial Registration:**

ClinicalTrials.gov, identifier NCT00656864.

## Introduction

Pioglitazone has been widely used for the treatment of type 2 diabetes (T2D) for its potent insulin sensitizing effect that preserves beta-cell function and causes durable reduction in HbA1c, amid some negative side effects such as weight gain, fluid retention, and increased fracture risk (1–6). Recently, its cardiovascular risk benefits have been highlighted (7–9). Although pioglitazone’s mechanisms of action *via* peroxisome proliferator-activated receptor-γ (PPARγ) is well known, little to nothing is known about how pioglitazone influences systemic interorgan communication *via* extracellular vesicles (EVs). EVs are small particles, ranging in size from 30-400 nm, that are released from most cell types (10). EVs contain a variety of cargo molecules, including microRNAs, mRNAs, proteins, and lipids, reflecting the cell of origin as well as its physiological state. Adipose tissue (AT) has been identified as a major source of circulating EV microRNAs (EV-miRNAs) (11). EVs and EV-miRNAs mediate crosstalk between cells within and between tissues as well as between organs in both health and disease, including diabetes (12–14). EVs are, thus, potential effectors of metabolic responses to pharmacologic agents for the treatment of diabetes.

Pioglitazone is a potent agonist of PPARγ, which is predominantly expressed in AT and is a master regulator of adipogenesis (15). Interestingly, PPARγ agonism has been shown to alter cancer derived EV-miRNAs (16), but it is not known whether pioglitazone treatment in people with T2D exerts any pharmacologic effect *via* regulation of AT-derived EV-miRNAs. To a lesser extent, pioglitazone also activates PPARα, which is expressed in many metabolically active tissues, is required for ketogenesis in the liver, and reduces inflammation by repressing endothelial TNFα-induced genes (17). Although pioglitazone treatment induces weight gain, a shift from visceral to subcutaneous fat distribution occurs and this shift associates with improvements in insulin sensitivity in the liver and peripheral tissues (18). We hypothesized that pioglitazone exerts systemic effects *via* the modulation of AT-derived EV cargo (i.e., EV-miRNAs) and that these changes would be related to changes in metabolic function in subjects with T2D.

## Materials and methods

### Clinical trial design and subjects.

The parent clinical trial (NCT00656864) was previously described (19). Twenty-four participants with well-controlled T2D (HbA1C<7%) on diet and exercise or a stable dose of metformin were recruited for a randomized, double-blind, and placebo-controlled study. Subjects with well-controlled T2D were selected to limit the confounding effect of glucotoxicity (the deleterious effects of chronic hyperglycemia on β-cell function). Female subjects were postmenopausal and subjects taking metformin continued their metformin treatment throughout the study. Subjects underwent a standard 75g oral glucose tolerance test (OGTT) at baseline and after 12 weeks of treatment with either placebo (PLA, n=12) or pioglitazone (PIO, 45 mg/day; n=12). The study had over 80% power to detect 1.5-fold change difference in miRNA expression using a two-sided test and significance level of 0.05. All aspects of the studies were approved by the Institutional Review Boards of the University of Vermont. All study participants provided written informed consent prior to participation.

### Blood analyses

Fasting blood samples were collected after an overnight fast in tubes containing EDTA or in serum-separating tubes. Plasma and serum samples were frozen at -80°C until analyses. HbA1C was measured on a COBAS INTEGRA 800 (Roche Diagnostics, Mannheim, Germany) automated analyzer. Insulin, C-peptide, and TNFα were measured by immunoassay (Insulin ELISA from Alpco, Salem, NH and MesoScale Discovery, Rockville, MD). Blood glucose concentrations were measured using YSI 2300 Stat Plus Glucose/Lactate Analyzer (YSI, Yellow Springs, OH). Data from an insulin-modified intravenous glucose tolerance test (IVGTT) (19) was used to calculate insulin sensitivity (Si), acute insulin response to glucose (AIRg), and glucose effectiveness (Sg) using the Minimal Model method of Bergman (20). Free fatty acids (FFA) were measured using the HR Series NEFA-HR (2) (Wako, Mountain View, CA) following the manufacturer’s instructions. The rate of FFA disappearance (K_FFA_) was calculated as the slope of the regression line fitting the natural logarithm of FFA concentration between 19 and 40 min, multiplied by -100 (21). Adipo-IR for each time point was calculated by multiplying the fasting FFA concentration (mmol/L) by the fasting insulin concentration (pmol/L) (22, 23).

### Adipose tissue biopsy

Subcutaneous periumbilical abdominal adipose tissue was obtained by percutaneous needle biopsy under local anesthesia. Tissue was aspirated into sterile PBS and washed twice to remove any blood. Whole tissue samples were then snap frozen in liquid nitrogen and stored at -80°C.

### Extracellular vesicles isolation and characterization

Extracellular vesicles were isolated from a total of 500 µl of fasting plasma samples using the Total Exosome Isolation Kit (from plasma) (ThermoFisher) according to the manufacturer’s instructions. Exosomal protein (35µg) was resolved in 4–20% Criterion TGX Precast Midi Protein gels (Bio-Rad, Hercules, CA), transferred to nitrocellulose membranes (Bio-Rad), and incubated overnight at 4°C with primary antibodies anti-CD9 (C-4) (Santa Cruz Biotechnology, sc-13118), anti-CD63 (H-193) (Santa Cruz Biotechnology, sc-15363), and anti-Calnexin (H-70) (Santa Cruz Biotechnology, sc-11397). Membranes were then incubated in appropriate species-specific secondary antibodies for 1h (IRDye 800CW anti-Rabbit IgG No. 926-32211 and IRDye 680RD anti-Mouse IgG No. 926-68070; Li-Cor Biosciences, Lincoln, NE). Protein bands were visualized using a Li-Cor Odyssey infrared imaging system (Li-Cor Biosciences). Quantification of extracellular vesicle concentration was conducted using the CD9 ExoELISA Complete Kit from Systems Biosciences Inc (Palo Alto, CA) according to manufacturer’s instruction. Extracellular vesicle samples for Transmission Electron Microscopy (TEM) were fixed in 2% paraformaldehyde, deposited on Formvar-carbon coated electron microscopy grids and contrasted and embedded first with a solution of 2% uranyl acetate then in 0.4% uranyl acetate in 2% methyl cellulose. TEM was performed using a FEI Morgagni TEM equipped with Gatan camera and microscope suite software. Nanoparticle Tracking Analysis (NTA) was performed with a NanoSight NS300 instrument with NTA-3.4 software (Malvern Panalytical, Malvern, UK). Five standard measurements of 1 minute each were taken at 25°C using continuous syringe pump speed. Camera level was set to 12 and detection threshold to 5 for all sample measurements.

### Profiling of miRNA abundance in circulating EVs

Total RNA from isolated extracellular vesicles was purified using Qiagen miRNeasy micro kit (Qiagen; Valencia, CA). miRNA expression was profiled by RT-PCR using custom designed 48-feature TaqMan MicroRNA Array cards and ViiA-7 instrument from ThermoFisher Scientific (Waltham, MA), following the manufacturer’s instructions. Forty-two miRNAs, previously reported to be expressed in adipocyte-derived extracellular vesicles, were included in the custom panel (**Supplementary Table ST2**). Raw Ct data was first adjusted based on the recovery of spike-in cel-miR-39, then normalized against the geometric mean of the top, most stable pair of miRNAs (miR-126 and miR-30b) identified by the NormFinder algorithm. Features with less than 1.5-fold change were filtered out. Filtered log fold ratio data was used for differential expression analysis as described in the Statistical Analysis section.

### Expression profiling of select miRNAs and miRNA-overtargeted genes in human adipose tissue

Adipose tissue RNA was extracted using RNeasy Lipid Tissue Mini Kit (Qiagen) with RNase-Free DNase treatment (Qiagen), following the manufacturer’s instructions. A cDNA library was made using Advantage-RT-for-PCR kits (Clontech; Mountain View, CA) using 1μg RNA template and oligo-dT primers. Gene expression was measured by quantitative real-time PCR using TaqMan reagents (**Supplementary Table ST4**). Relative expression levels were calculated using the -ΔΔCt method. Adipose tissue miRNA data was normalized against endogenous control miR-191. Adipose tissue gene expression data was normalized against housekeeping gene PPIB.

### Functional network analysis

We conducted miRNA-overtargeted network analyses following our published methodology (24). Significance of cooperative and overtargeting effects were assessed by simulating 10,000 equivalent random networks. The list of validated targets supported by strong experimental evidence (i.e., reporter assay or Western blot) used for this analysis was downloaded from miRTarBase (file: miRTarBase_SE_WR.xls) using the SpidermiR package (25). Interaction networks were constructed using Cytoscape 3.5.1 (26). Enrichment of gene ontology annotations among sets of overtargeted genes was assessed using the GOCluster_Report function of the systemPipeR package (27) in the R environment.

### Statistical analysis

Differences in baseline clinical characteristics were assessed using the TableOne R package and the nonparametric Mann-Whitney U test (for continuous variables) or the Fisher exact test (for categorical variables). For assessment of longitudinal differences in clinical, metabolic, circulating miRNA, and AT-gene variables, mixed-effect models for repeated measures were implemented using the nlme package. Measurements done in circulation were adjusted for body weight and hematocrit measurement to account for the potential confounding effects of weight gain and increased blood volume known to be induced by pioglitazone. Measurements of adipose tissue gene expression were adjusted for body weight to account for the potential confounding effect of weight gain (the pioglitazone-induced increase in blood volume was considered to be a confounding factor only for variables measured in plasma or serum). Subject ID was included as a random effect variable to account for the correlations between repeated measurements within each patient. To ensure that the use of metformin by a subgroup of participants did not affect the results obtained with our minimal models, these analyses were repeated including metformin use as an additional cofactor. However, because the study groups are well balanced and the sample size of the study is relatively small, we had *a priori* decided to include a minimal number of variables in the final models. Partial correlations were also calculated in the R environment adjusting for the same covariates. *Post-hoc* analysis was performed using the phia package. Calculated effects and correlations with two-tailed P.value<0.05 were considered significant. False discovery rates (FDR) correcting for multiple testing were calculated using the Benjamini-Hochberg (BH) correction. Based on the well-established role of miRNAs in repression of target genes (28, 29), our analysis of expression changes in miRNA-overtargeted genes in human adipose tissue *a priori* considered that upregulated AT-miRNAs would cause downregulation of overtargeted genes. Therefore, a one-sided P.value<0.05, was considered significant in those cases.

## Results

### Concentration of circulating EVs does not significantly change as quantified by NTA and CD9 ELISA

In this longitudinal study, we used archived plasma and subcutaneous adipose tissue samples from a 3-month randomized, controlled trial (NCT00656864). Participants included 24 subjects with T2D, who were randomized to treatment with either pioglitazone 45 mg/day (PIO, n=12) or placebo (PLA, n=12). The two groups were balanced with respect to most baseline clinical measures (**Supplementary Table ST1**). As reported in the parent study (19), treatment with pioglitazone increased weight and BMI and was associated with significant improvements in insulin sensitivity and HbA1c levels.

**Figure 1:**
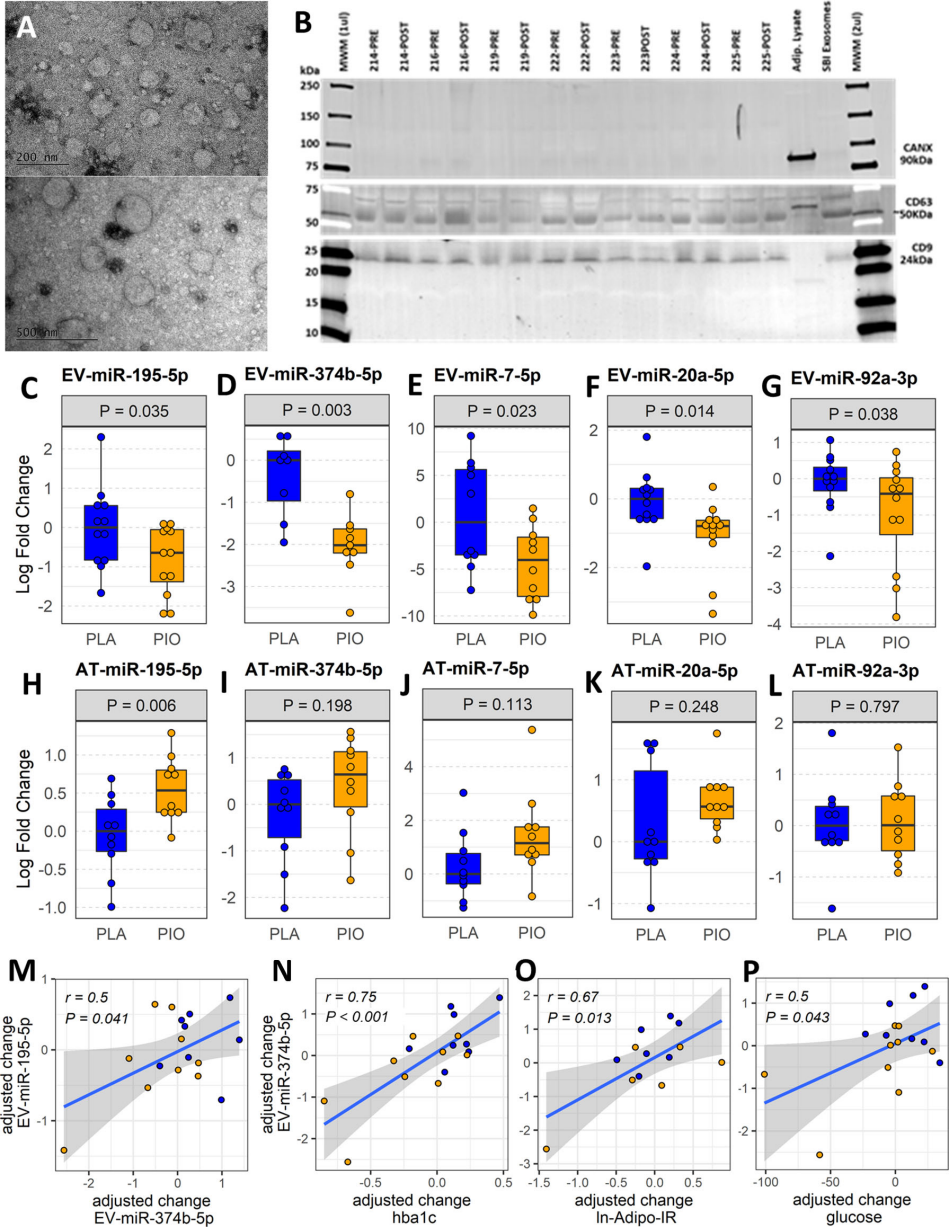
Profiling plasma EV-miRNAs and AT-miRNAs in subjects with T2D treated with pioglitazone (PIO, yellow color filling) or placebo (PLA, blue color filling). **(A)** Transmission electron micrographs of representative EV preparation. **(B)** Western blot for exosomal (CD9, CD63) and non-exosomal (CANX) protein markers. Blots show samples from 7 out of the 12 patients from both groups, prior and after treatment (no significant differences found by densitometry quantification of protein band content). Adipocyte lysate and SBI Exosome lysate were used as controls. **(C-G)** Baseline-adjusted boxplots of five significantly downregulated miRNAs in circulating EVs from T2D subjects treated with PIO, compared to PLA. **(H-L)** Baseline-adjusted boxplots of miRNA expression in adipose tissue (AT) from T2D subjects treated with PIO, compared to PLA group. **(M-P)** Significant partial correlations between EV-miRNAs and relevant clinical measures.

EVs were isolated from fasting plasma samples collected before and after the 12-week treatment period. The characterization of the EV preparations by TEM and Western blot (**Figures 1A, B**) showed an enrichment in nanoparticles with sizes, shapes, and membrane protein markers that were compatible with those of exosomes and small microvesicles (10). In addition, these preparations were free of the non-exosomal marker calnexin (**Figure 1B**). Neither PIO nor PLA treatment significantly changed the average number of circulating EVs in the study subjects, as quantified by NTA and CD9 ELISA (**Supplementary Figure SF1**).

### Five miRNAs were downregulated in circulating EVs after treatment with pioglitazone

The abundance of 42 miRNAs (**Supplementary Table ST2**) reported to be expressed in adipocyte-derived EVs (11) was profiled in EVs isolated from fasting plasma obtained before and after treatment. The levels of five circulating EV-miRNAs (*i.e.*, EV-miR-374b-5p, EV-miR-195-5p, EV-miR-20a-5p, and EV-miR-7-5p, and EV-miR-92a-3p) were significantly downregulated (fold change<-1.5, *P*<0.05, FDR ≤ 0.15) in response to PIO as compared to PLA treatment (**Figures 1C-G**, **Supplementary Table ST3-A**). The change in EV-miR-374b-5p significantly correlated with the change in EV-miR-195b-5p (r=0.5, P=0.041, FDR=0.11, **Figure 1M**). The change in EV-miR-374b-5p strongly correlated with the change in HbA1c, fasting plasma glucose, and insulin resistance in the adipose tissue (r≥0.5, P<0.05, FDR<0.1, **Figures 1N-P**), among others. Interestingly, hsa-miR-20a-5p and hsa-miR-92a-3p are located in the hsa-mir-92a-1 genomic cluster with an inter-miRNA distance smaller than 10kb (miRbase.org). These results were not affected by the inclusion of metformin use as an additional cofactor in the mixed-effect models.

### miR-195-5p is upregulated in subcutaneous adipose tissue after treatment with pioglitazone

**Figure 2:**
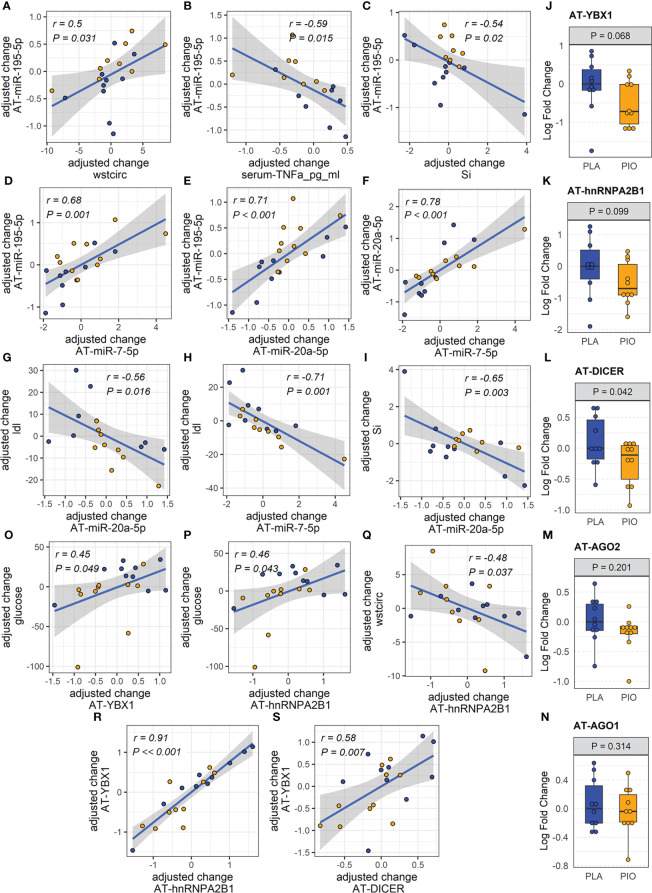
miRNAs and exosome sorting genes in AT. **(A-I)** Significantly correlated changes between DE AT-miRNAs and between DE AT-miRNAs and relevant clinical and biochemical measures. **(J-N)** Expression of AT transcripts involved in miRNA exosomal sorting and miRNA biogenesis. **(O-S)** Significantly correlated changes between AT genes involved in miRNA exosomal sorting and relevant clinical measures. Yellow color filling for pioglitazone group, blue color filling for placebo group.

Considering that AT is one of the main sites of pioglitazone action and that AT contributes a majority of EV-miRNAs into the circulation (11), we quantified the respective miRNA expression levels of the differentially expressed EV-miRNAs in subcutaneous AT samples from the same participants. In contrast to the downregulation of circulating EV-miRNAs, the expression of the corresponding miRNAs in AT (AT-miRNAs) was unchanged or increased in response to pioglitazone relative to placebo (**Figures 1H-L**, **Supplementary Table ST3-B**). Only miR-195-5p showed a statistically significant (P=0.006, FDR=0.03, **Figures 1H-L**) increase in AT and this change was significantly correlated with the change in waist circumference (r=0.76, P<0.018, FDR<0.18) and negatively correlated with the change in serum concentration of TNFα and in the insulin sensitivity index (Si) (-r>0.5, P<0.02, FDR<0.1) (**Figures 2A-C**). Additionally, the changes in AT-miR-195-5p, AT-miR-7-5p, and AT-miR-20a-5p expression levels were strongly correlated (all r>0.65, P<0.01, FDR<0.025, **Figures 2D-F**) and the changes in AT-miR-7-5p and AT-miR-20a-5p further correlated with the change in circulating LDL and Si, an index of insulin sensitivity (all r>0.55, P<0.02, FDR<0.05, **Figures 2G–I**). These results were not affected by the inclusion of metformin use as an additional cofactor in the mixed-effect models.

### Dicer, YBX1, and hnRNPA2B1 are downregulated in subcutaneous fat tissue from subjects with type 2 diabetes treated with pioglitazone

We reasoned that miRNAs upregulated in the AT (i.e., miR-195-5p) may be retained in the tissue by being diverted from the exosomal sorting pathway. To test this hypothesis, we evaluated potential causes of the reduced levels of circulating AT-derived EV-miRNAs in response to PIO. Using subcutaneous AT samples from the same participants, we quantified the expression of genes involved in miRNA biogenesis and/or sorting to the exosomal pathway (*i.e.*, Dicer, Ago2, YBX1, hnRNPA2B1 and nSMase2/SMPD3) (30). Because levels of AT-miRNAs increased in the PIO-treated group, we hypothesized that biogenesis-related Dicer and/or Ago2 would be upregulated in the AT of these participants. Because the miRNA levels were reduced in the plasma EVs of the PIO-treated subjects, we further hypothesized that exosomal sorting-related YBX1, hnRNPA2B1, and/or SMPD3 would be downregulated in the AT. We observed a trend for downregulation of AT-YBX1 (P=0.068, FDR=0.19) and AT-hnRNP2AB1 (P= 0.099, FDR=0.24) in response to pioglitazone treatment (**Figures 2J, K**). However, contrary to our expectations, AT-Dicer was significantly downregulated in the PIO group (P=0.042, FDR=0.19, **Figure 2L**). No significant changes were observed in Ago2 expression or the related family member Ago1 (**Figures 2M, N**). Importantly, changes in AT-YBX1 and AT-hnRNPA2B1 significantly correlated with the pioglitazone-induced changes in fasting glucose and waist circumference (absolute |r|≥0.45, P<0.05, FDR<0.08, **Figures 2O-Q**, **Supplementary Figure SF1**). Positive correlations were also observed between the change in AT-YBX1 and AT-DICER as well as the change in AT-YBX1 and AT-hnRNPA2B1 (r≥0.58, P ≤ 0.007, FDR ≤ 0.03, **Figures 2R, S**). These results were not affected by the inclusion of metformin use as an additional cofactor in the mixed-effect models.

### A network of miRNA-overtargeted genes are downregulated in subcutaneous adipose tissue from subjects with type 2 diabetes treated with pioglitazone

**Figure 3:**
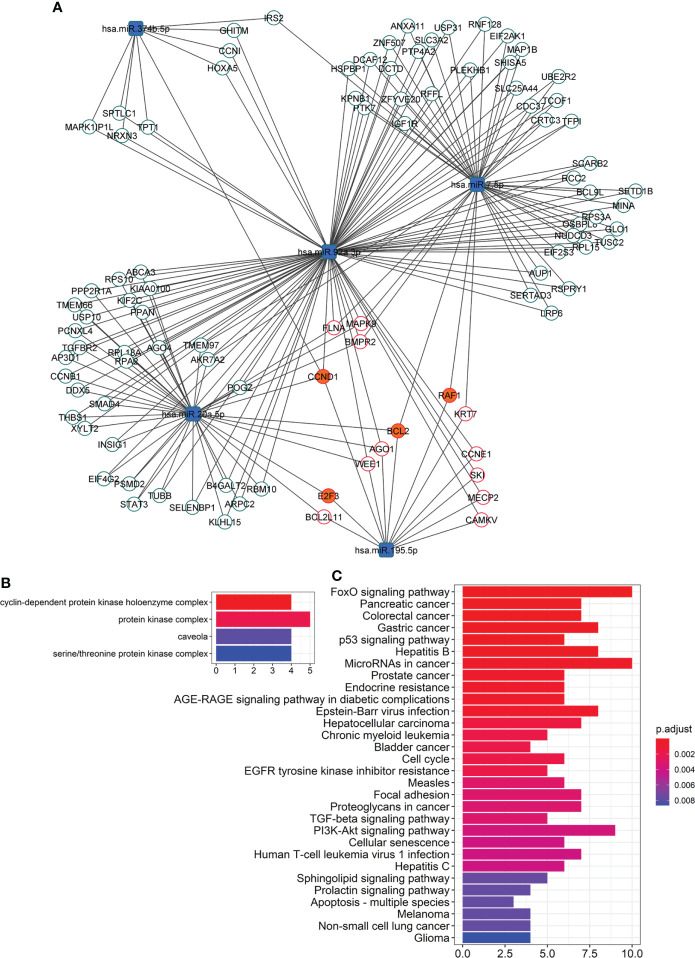
Network of overtargeted miRNA-mRNA interactions. **(A)** This particular type of network, only includes transcripts validated to interact with the differentially expressed DE miRNAs that were found to be targeted by more DE miRNAs than expected by chance (“miRNA-overtargeted”). **(B)** Cellular compartments enriched among the annotations of the miRNA-overtargeted genes. **(C)** KEGG pathways enriched among the annotations of the miRNA-overtargeted genes.

To further evaluate the functional relevance of the altered EV-miRNAs in response to pioglitazone treatment, we built a network of validated miRNA-gene interactions that identified 96 transcripts as overtargeted (i.e., targeted by more differentially expressed EV-miRNAs than expected by chance, **Figure 3A**, *P_simulation_* =0.0052). The analysis shown in **Figures 3B, C** highlights the top cell compartments and KEGG pathways enriched in the overtargeted gene network, respectively. The caveola was found to be a key EV-miRNA-targeted compartment in response to pioglitazone treatment. On the other hand, the enrichment in overtargeted KEGG pathways highlighted miRNA-regulated pathways in cancer, resistance to endocrine therapy, FoxO signaling, PI3K/Akt signaling, and sphingolipid signaling, among others (**Figure 3C**).

**Figure 4:**
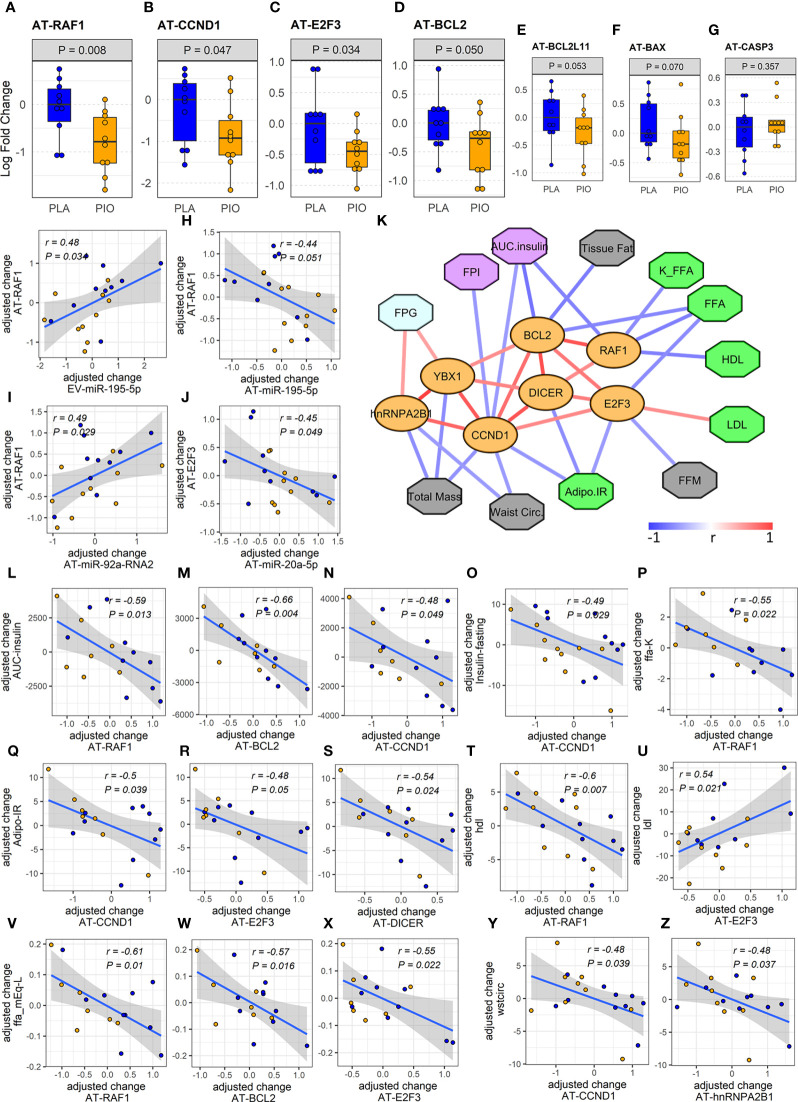
Expression and associations among AT genes found to be overtargeted by the DE EV-miRNAs. **(A-E)** Profiles of differentially expressed overtargeted AT genes. **(F, G)** Profiles of pro-apoptotic BAX and CASP3 genes in AT. **(G-J)** Significant correlations between the change in DE overtargeted AT genes and the change in circulating DE EV-miRNA and AT-miRNAs. **(K)** Correlation network of significant (*P*<0.05, FDR<0.1) associations among AT genes involved in miRNA biogenesis/exosomal sorting, experimentally validated AT genes overtargeted by the DE EV-miRNAs (orange ovals), and clinical measures relevant to lipid metabolism (green octagons), body composition (grey octagons), glycemic control (cyan octagons), and β cell function (purple octagons). **(L-Z)** Correlation plots between the change in DE AT genes and the change in relevant clinical measures. Waist circ or wstcirc, waist circumference; FPG, fasting plasma glucose; FPI, fasting plasma insulin; AUC.insulin, insulin area under the curve; FFM, free fat mass; FFA, free fatty acids; K_FFA or ffa.K, rate of free fatty acid disappearance; LDL, low density lipoprotein; HDL, high density lipoprotein; Adipo.IR, insulin resistance in adipose tissue.

To confirm the functional relevance of this miRNA-overtargeted gene network in a specific human tissue, we quantified the expression of overtargeted genes (specifically those validated as interacting with miR-195-5p as well as 3 other genes not targeted by miR-195-5p but central to the network by interacting with 3 of the 5 differentially abundant EV-miRNAs) in subcutaneous AT from the same study participants. Remarkably, the expression levels of 4 transcripts (*i.e.*, RAF1, CCND1, BCL2, and E2F3) were significantly reduced in AT (**Figures 4A-D**) and strongly correlated with changes in targeting miRNAs (**Figures 4G-J**). These miRNA-overtargeted genes additionally displayed strong co-regulation in AT as indicated by highly significant correlated changes among each other (**Figure 4K**; **Supplementary Table ST4**). Their changes also significantly correlated with the change in differentially expressed Dicer and the exosomal miRNA-sorting genes YBX1 and hnRNPA2B1 in the AT (**Figure 4K**, **Supplementary Table ST4**). Most importantly, the change in these pioglitazone-associated AT genes significantly correlated with the change in key clinical measures of insulin secretion and function (fasting plasma insulin –FPI, insulin AUC, and insulin resistance in AT –Adipo IR), lipid metabolism (rate of free fatty acids disappearance –K_FFA, serum FFA concentration, HDL, and LDL), glycemic control (fasting plasma glucose –FPG), and body composition (waist circumference, tissue fat, free fat mass, and total mass) (absolute |r|≥0.45, P<0.05, FDR<0.1, **Figures 4L-Z**). These results were not affected by the inclusion of metformin use as an additional cofactor in the mixed-effect models.

## Discussion

The observed correlation between the change in EV-miR-374b-5p and EV-miR-195-5p suggests the coregulation of these EV-miRNAs. The strong associations of EV-miR-374b-5p changes with changes in HbA1c, fasting plasma glucose, and insulin resistance in the adipose tissue indicate that plasma EV-miRNAs are functionally associated with the glycemic control exerted by the pioglitazone treatment. On the other hand, the strong correlations among the changes in AT-miR-195-5p, AT-miR-7-5p, and AT-miR-20a-5p expression levels and with the changes in levels of circulating LDL and the insulin sensitivity index also suggest the coregulation of transcriptional mechanisms leading to the production of these miRNAs in AT. In particular, the pioglitazone-associated reduction in miR-195-5p in circulating EVs and corresponding increased expression in AT appear to contribute to improved inflammatory milieu, glycemic control, and insulin sensitivity in subjects with T2D. Interestingly, we previously reported that circulating miR-195-5p and miR-7-5p were reduced by short-term intensive insulin therapy in subjects with early T2D (24) and vitamin D supplementation in subjects with prediabetes (31), respectively. Collectively, these findings suggest general roles for EV-miR-195-5p and EV-miR-7-5p, in response to distinct anti-hyperglycemic treatments. Other authors have reported that miR-7-5p induces repression of β cell function and proliferation (32) and also plays a crucial role as PPARα-regulated mediator of lipid metabolism in hepatocytes, where it activates SREBP signaling and promotes lipid accumulation (33). Further supporting our findings, a study by Papi and colleagues reported that pioglitazone can abolish the capability of hypoxic breast cancer cell-derived exosomes to induce miRNA-mediated pro-inflammatory and pro-invasive pathways in surrounding tumor associated fibroblasts (16). We reason that, by analogy (i.e., modifying the miRNA content of secreted EVs), pioglitazone might mitigate the capacity of EVs to induce miRNA-mediated diabetogenic changes in metabolism.

Interestingly, we demonstrate that the upregulation of pioglitazone-responsive miRNAs in AT does not occur because of increased Dicer/Ago2-mediated miRNA biogenesis but likely, at least in part, because of suppression of miRNA secretion *via* the exosome pathway. The correlations between the changes in AT-YBX1 and AT-hnRNPA2B1 with the pioglitazone-induced changes in fasting glucose and waist circumference support our reasoning. Collectively, these results indicate that pioglitazone treatment alters the sorting of specific miRNAs into the EV compartment of AT in people with T2D, and this is related to the drug-induced improvements in glycemic control and body fat redistribution.

The analysis highlighting the top cell compartments and KEGG pathways enriched in the miRNA-overtargeted gene network identified the caveola, a cell membrane compartment involved in cellular trafficking and signaling and in lipid turnover (34), as a key EV-miRNA-targeted compartment in response to pioglitazone treatment. Deficiency in this compartment was reported to cause maladaptative autophagy that contributes to insulin resistance and altered adipocyte differentiation (35). The enrichment in KEGG pathways such as FoxO signaling, PI3K/Akt signaling, and sphingolipid signaling is also consistent with a broad array of signaling involved with diabetes and its complications. The recurrent link with cancer and the response to anticancer therapies suggests that pioglitazone affects conserved biochemical pathways contributing to both, diabetes and cancer. Specifically, the well-established role for phosphatidylinositol triphosphate (PI (3–5)P3) signaling to regulate insulin sensitivity and cell metabolism *via* modulation of Akt activity (36, 37), as well as tumor growth (38, 39) and migration of T lymphocytes *via* modulation of FoxO signaling (40, 41), among others, underscore the mechanistic impact of pioglitazone treatment in the modulation of metabolism, inflammation, and the tumor microenvironment. The miRNA-targeting of sphingolipid signaling is also important and likely related to the mechanisms by which pioglitazone restores the altered lipid metabolism in diabetes. Supporting this reasoning, profiling of circulating sphingolipids uncovered that pioglitazone treatment in people with metabolic syndrome induced a potent decrease in plasma ceramides and that some of those changes correlated with changes in adiponectin levels and insulin resistance (42, 43).

Among the miRNA-overtargeted genes, RAF1 demonstrated the most significant downregulation in the subcutaneous AT of the PIO group (**Figure 4A**). Interestingly, RAF1 is a known positive regulator of lipolysis in adipocytes (44). We reason that, by targeting RAF1, the pioglitazone-induced elevation of AT-miR-195-5p may enhance the suppression of lipolysis, leading to weight gain but also improved insulin sensitivity. Suppression of lipolysis was also suggested by the associations we detected between the changes in AT-miRNAs and K_FFA and Adipo-IR. Downregulation of RAF1 in AT could additionally contribute to antioxidant effects of pioglitazone with concomitant reduction of cellular stress (45). On the other hand, cyclin D1 (CCND1), another significantly downregulated miRNA-overtargeted gene in our study, was reported to inhibit mitochondrial function and size and its antisense inactivation shown to enhance oxidative glycolysis and lipogenesis (46). These findings suggest that CCND1 downregulation in AT may contribute to pioglitazone-induced weight gain. Interestingly, we now also report that changes in CCND1, RAF1, and E2F3 negatively correlate with the change in insulin AUC and in fasting plasma insulin (**Figures 4K, N, O**), which suggests an association between the expression of these pioglitazone-responsive miRNA-regulated genes in AT and insulin secretion from the β cells. This further suggests the existence of a modifiable adipose–pancreas interorgan communication axis that may involve circulating EVs with drug-inducible cargo modifications.

On the other hand, the significant downregulation of the miRNA-overtargeted anti-apoptotic gene BCL2 in AT from the PIO group seemed counterintuitive. However, in the context of a reduction of pro-apoptotic factors due to a presumable improvement of the inflammatory milieu, the compensatory reduction of anti-apoptotic factors seems plausible. Supporting this idea, the overtargeted pro-apoptotic gene BCL2L11 also demonstrated a trend toward downregulation (P=0.053, FDR=0.19, **Figure 4E**). To further assess our hypothesis, we additionally measured the expression of death-promoting BAX and CASP3 genes in AT. BAX demonstrated a trend (*P*=0.07, FDR=0.19) towards downregulation with no significant change in CASP3 levels (**Figures 4F, G**). Notably, the changes in AT-BCL2 and AT-BAX were significantly correlated with each other, negatively correlated with the change in AUC insulin, and tended to positively correlate with the change in circulating TNFα (**Supplementary Figure SF1**). We reason that the decrease in AT expression of these stress/death-related transducers, together with the downregulation of CCND1 and E2F3 [known effectors of cytokine signaling and cellular proliferation (47)], may represent an improvement in the basal metabolic and anti-inflammatory milieu in pioglitazone-treated subjects.

The effects of reduced miR-195-5p in the circulating EVs could additionally and beneficially impact biological process on distant organs such as the liver and heart. In the liver, miR-195-5p could activate hepatic stellate cells (48). Therefore, reduced circulating EV-miR-195-5p could contribute to reduced liver fibrosis [as observed after pioglitazone treatment in a model of rat liver cirrhosis (49)] and/or reduced liver steatosis, inflammation, and ballooning [as observed after pioglitazone treatment in NAFLD patients with prediabetes or T2D (50)]. On the other hand, in rat models of diabetic cardiomyopathy (DCM), inhibition of the elevated levels of miR-195-5p reduced cardiac dysfunction, myocardial fibrosis, collagen deposition, and endothelial mesenchymal transition (51). Thus, the reduced circulating EV-miR-195-5p after pioglitazone treatment in humans with T2D could mechanistically contribute to the improved cardiovascular outcomes observed after treatment with the drug in these patients (52).

Although our study provides insight into novel mechanisms potentially driving and/or regulating the anti-diabetic effects of pioglitazone, several limitations need to be acknowledged. First, the relatively small sample size of the study is one key limitation that likely affected our power to detect significant changes in several of the measured variables. For example, the changes in AT miRNAs miR-374b-5p and miR-7-5p, and AT genes YBX1 and hnRNPA2B1 did not reach the standard significance level possibly due to the limited power of our study. Supporting our reasoning, most of these features displayed important trends with P values < 0.1 and the changes in several of them demonstrated significant correlations with the changes in measures of body composition, glycemic control, beta cell function, and insulin action. We believe that part of the relevance of our study is in the power of the combined results, which elicit important “network effects” that support a role for these molecules in modulating the response to pioglitazone treatment. Another limitation of this study is that, although access to paired adipose tissue from the same study participants was important to develop our working model, assessment of the differentially expressed miRNAs and their target genes in other tissues such as liver, pancreas, and muscle is required for a comprehensive assessment of EV-mediated mechanisms driving interorgan communication in response to anti-diabetic drugs. Finally, validation of our findings in an independent study cohorts is necessary to confirm and ensure generalizability of our results.

## Conclusions

In this study, we found that the levels of five miRNAs reportedly derived from AT, namely miR-7-5p, miR-20a-5p, miR-92a-3p, miR-195-5p, and miR-374b-5p, were significantly downregulated in plasma EVs in response to pioglitazone treatment in people with T2D. Interestingly, downregulation of miR-195-5p in the plasma EVs contrasted with increased levels of the miRNA in AT. Downregulation of DICER and exosomal miRNA-sorting-related genes YBX1 and hnRNPA2B1 in AT suggests that miR-195-5p may accumulate in adipose tissue not because increased miRNA biogenesis but due to reduced exosomal sorting and disposal. Importantly, changes in the abundance of EV-miRNAs and AT-miRNAs correlated with changes in measures of glycemic control, β cell function, and lipid metabolism. These miRNAs overtargeted a network of transcripts that changed in AT in an apparently coordinated manner. Among key miRNA overtargeted and differentially expressed AT-genes, downregulated RAF1 may contribute to insulin-independent suppression of AT lipolysis, while downregulated CCND1 may contribute to enhanced mitochondrial function and lipogenesis in response to pioglitazone treatment. These events may contribute to the known pioglitazone-induced weight gain with concomitant improvement of insulin sensitivity and glucose homeostasis. Although additional studies are needed for confirmation, our study underscores the potential role of adipose-derived EV-miRNAs in the regulation of fat redistribution and insulin function in people with type 2 diabetes undergoing pioglitazone treatment.

## Data availability statement

The raw data supporting the conclusions of this article will be made available by the authors, without undue reservation.

## Ethics statement

The studies involving human participants were reviewed and approved by Institutional Review Boards of the University of Vermont. The patients/participants provided their written informed consent to participate in this study.

## Author contributions

YNL conducted experiments, researched data, and wrote the manuscript. AC researched data, contributed to discussions, and reviewed/edited the manuscript. ZK and AMP conducted experiments, contributed to discussions, and reviewed/edited the manuscript. OS contributed to discussions and reviewed/edited the manuscript. WGT researched data, contributed to discussions and reviewed/edited the manuscript. REP designed the study, acquired funding, provided the study samples, supervised the work, researched data, contributed to discussions, and reviewed/edited the manuscript. All authors contributed to the article and approved the submitted version.

## Funding

This study was funded by program funds granted to REP by the AdventHealth Translational Research Institute.

## Acknowledgments

The parent clinical trial was funded by Takeda Pharmaceuticals North America.

## Conflict of interest

This study was funded by program funds granted to RP by the AdventHealth Translational Research Institute. The parent clinical trial was supported by an investigator-initiated grant to RP from Takeda Pharmaceuticals North America. RP reports grants from Hanmi Pharmaceutical Co.; grants from Janssen; consulting fees from Merck; grants, speaker fees and consulting fees from Novo Nordisk; consulting fees from Pfizer; grants from Poxel SA; grants and consulting fees from Sanofi; consulting fees from Scohia Pharma Inc.; consulting fees from Sun Pharmaceutical Industries. AC reports consulting fees from GlaxoSmithKline. Honoraria and fees for RP’s and AC’s services were paid directly to AdventHealth, a nonprofit organization. No other potential conflicts of interest relevant to this article were reported by any of the other authors.

## Publisher’s note

All claims expressed in this article are solely those of the authors and do not necessarily represent those of their affiliated organizations, or those of the publisher, the editors and the reviewers. Any product that may be evaluated in this article, or claim that may be made by its manufacturer, is not guaranteed or endorsed by the publisher.

## References

[1] Defronzo RA Mehta RJ Schnure JJ . Pleiotropic effects of thiazolidinediones: implications for the treatment of patients with type 2 diabetes mellitus. Hosp Pract (1995) 41(2013):132–47. doi: 10.3810/hp.2013.04.1062 23680744

[2] Belfort R Harrison SA Brown K Darland C Finch J Hardies J . A placebo-controlled trial of pioglitazone in subjects with nonalcoholic steatohepatitis. N Engl J Med (2006) 355:2297–307. doi: 10.1056/NEJMoa060326 17135584

[3] Triplitt C Cersosimo E DeFronzo RA . Pioglitazone and alogliptin combination therapy in type 2 diabetes: a pathophysiologically sound treatment. Vasc Health Risk Manag (2010) 6:671–90. doi: 10.2147/vhrm.s4852 PMC2941781 20859539

[4] Bajaj M Suraamornkul S Piper P Hardies LJ Glass L Cersosimo E . Decreased plasma adiponectin concentrations are closely related to hepatic fat content and hepatic insulin resistance in pioglitazone-treated type 2 diabetic patients. J Clin Endocrinol Metab (2004) 89:200–6. doi: 10.1210/jc.2003-031315 14715850

[5] Gross B Staels B . PPAR agonists: multimodal drugs for the treatment of type-2 diabetes. best practice & research. Clin Endocrinol Metab (2007) 21:687–710. doi: 10.1016/j.beem.2007.09.004 18054742

[6] Inzucchi SE Viscoli CM Young LH Furie KL Gorman M Lovejoy AM . Pioglitazone prevents diabetes in patients with insulin resistance and cerebrovascular disease. Diabetes Care (2016) 39:1684–92. doi: 10.2337/dc16-0798 PMC5033078 27465265

[7] DeFronzo RA Inzucchi S Abdul-Ghani M Nissen SE . Pioglitazone: The forgotten, cost-effective cardioprotective drug for type 2 diabetes. Diabetes Vasc Dis Res (2019) 16:133–43. doi: 10.1177/1479164118825376 30706731

[8] Nesti L Tricò D Mengozzi A Natali A . Rethinking pioglitazone as a cardioprotective agent: a new perspective on an overlooked drug. Cardiovasc Diabetol (2021) 20:109. doi: 10.1186/s12933-021-01294-7 34006325 PMC8130304

[9] Dormandy JA Charbonnel B Eckland DJ Erdmann E Massi-Benedetti M Moules IK . Secondary prevention of macrovascular events in patients with type 2 diabetes in the PROactive study (PROspective pioglitAzone clinical trial in macroVascular events): a randomised controlled trial. Lancet (2005) 366:1279–89. doi: 10.1016/S0140-6736(05)67528-9 16214598

[10] Raposo G Stoorvogel W . Extracellular vesicles: exosomes, microvesicles, and friends. J Cell Biol (2013) 200:373–83. doi: 10.1083/jcb.201211138 PMC3575529 23420871

[11] Thomou T Mori MA Dreyfuss JM Konishi M Sakaguchi M Wolfrum C . Adipose-derived circulating miRNAs regulate gene expression in other tissues. Nature (2017) 542:450–5. doi: 10.1038/nature21365 PMC5330251 28199304

[12] Freeman DW Noren Hooten N Eitan E Green J Mode NA Bodogai M . Altered extracellular vesicle concentration, cargo, and function in diabetes. Diabetes (2018) 67:2377–88. doi: 10.2337/db17-1308 PMC6198336 29720498

[13] Noren Hooten N Evans MK . Extracellular vesicles as signaling mediators in type 2 diabetes mellitus. Am J Physiol Cell Physiol (2020) 318:C1189–c1199. doi: 10.1152/ajpcell.00536.2019 32348178 PMC7311734

[14] Prattichizzo F Matacchione G Giuliani A Sabbatinelli J Olivieri F de Candia P . Extracellular vesicle-shuttled miRNAs: a critical appraisal of their potential as nano-diagnostics and nano-therapeutics in type 2 diabetes mellitus and its cardiovascular complications. Theranostics (2021) 11:1031–45. doi: 10.7150/thno.51605 PMC7738884 33391519

[15] Wang YX . PPARs: diverse regulators in energy metabolism and metabolic diseases. Cell Res (2010) 20:124–37. doi: 10.1038/cr.2010.13 PMC4084607 20101262

[16] Papi A De Carolis S Bertoni S Storci G Sceberras V Santini D . PPARgamma and RXR ligands disrupt the inflammatory cross-talk in the hypoxic breast cancer stem cells niche. J Cell Physiol (2014) 229:1595–606. doi: 10.1002/jcp.24601 24604522

[17] Orasanu G Ziouzenkova O Devchand PR Nehra V Hamdy O Horton ES . The peroxisome proliferator-activated receptor-gamma agonist pioglitazone represses inflammation in a peroxisome proliferator-activated receptor-alpha-dependent manner *in vitro* and *in vivo* in mice. J Am Coll Cardiol (2008) 52:869–81. doi: 10.1016/j.jacc.2008.04.055 PMC2633943 18755353

[18] Miyazaki Y Mahankali A Matsuda M Mahankali S Hardies J Cusi K . Effect of pioglitazone on abdominal fat distribution and insulin sensitivity in type 2 diabetic patients. J Clin Endocrinol Metab (2002) 87:2784–91. doi: 10.1210/jcem.87.6.8567 12050251

[19] Tharp WG Gupta D Sideleva O Deacon CF Holst JJ Elahi D . Effects of pioglitazone on glucose-dependent insulinotropic polypeptide–mediated insulin secretion and adipocyte receptor expression in patients with type 2 diabetes. Diabetes (2020) 69:146–57. doi: 10.2337/db18-1163 PMC13520063 31757794

[20] Pacini G Bergman RN . MINMOD: a computer program to calculate insulin sensitivity and pancreatic responsivity from the frequently sampled intravenous glucose tolerance test. Comput Methods Programs BioMed (1986) 23:113–22. doi: 10.1016/0169-2607(86)90106-9 3640682

[21] McLachlan KA Boston R Alford FP . Impaired non-esterified fatty acid suppression to intravenous glucose during late pregnancy persists postpartum in gestational diabetes: a dominant role for decreased insulin secretion rather than insulin resistance. Diabetologia (2005) 48:1373–9. doi: 10.1007/s00125-005-1775-6 15940468

[22] Gastaldelli A Harrison SA Belfort-Aguilar R Hardies LJ Balas B Schenker S . Importance of changes in adipose tissue insulin resistance to histological response during thiazolidinedione treatment of patients with nonalcoholic steatohepatitis. Hepatol (Baltimore Md.) (2009) 50:1087–93. doi: 10.1002/hep.23116 19670459

[23] Sondergaard E Espinosa De Ycaza AE Morgan-Bathke M Jensen MD . How to measure adipose tissue insulin sensitivity. J Clin Endocrinol Metab (2017) 102:1193–9. doi: 10.1210/jc.2017-00047 PMC5460729 28323973

[24] Nunez Lopez YO Retnakaran R Zinman B Pratley RE Seyhan AA . Predicting and understanding the response to short-term intensive insulin therapy in people with early type 2 diabetes. Mol Metab (2019) 20:63–78. doi: 10.1016/j.molmet.2018.11.003 30503831 PMC6358589

[25] Cava C Colaprico A Bertoli G Graudenzi A Silva TC Olsen C . SpidermiR: An R/Bioconductor package for integrative analysis with miRNA data. Int J Mol Sci (2017) 18:274. doi: 10.3390/ijms18020274 PMC5343810 28134831

[26] Demchak B Hull T Reich M Liefeld T Smoot M Ideker T . Cytoscape: the network visualization tool for GenomeSpace workflows. F1000Research (2014) 3:151. doi: 10.12688/f1000research.4492.2 25165537 PMC4133763

[27] Backman T Girke T . systemPipeR: NGS workflow and report generation environment. BMC Bioinf (2016) 17:388. doi: 10.1186/s12859-016-1241-0 PMC5029110 27650223

[28] Gebert LFR MacRae IJ . Regulation of microRNA function in animals. Nat Rev Mol Cell Biol (2019) 20:21–37. doi: 10.1038/s41580-018-0045-7 30108335 PMC6546304

[29] Bartel DP . Metazoan MicroRNAs. Cell (2018) 173:20–51. doi: 10.1016/j.cell.2018.03.006 29570994 PMC6091663

[30] Zhang J Li S Li L Li M Guo C Yao J . Exosome and exosomal microRNA: trafficking, sorting, and function. Genomics Proteomics Bioinf (2015) 13:17–24. doi: 10.1016/j.gpb.2015.02.001 PMC4411500 25724326

[31] Nunez Lopez YO Pittas AG Pratley RE Seyhan AA . Circulating levels of miR-7, miR-152 and miR-192 respond to vitamin d supplementation in adults with prediabetes and correlate with improvements in glycemic control. J Nutr Biochem (2017) 49:117–22. doi: 10.1016/j.jnutbio.2017.08.007 28945992

[32] Latreille M Hausser J Stutzer I Zhang Q Hastoy B Gargani S . MicroRNA-7a regulates pancreatic beta cell function. J Clin Invest (2014) 124:2722–35. doi: 10.1172/JCI73066 PMC4038573 24789908

[33] Singaravelu R Quan C Powdrill MH Shaw TA Srinivasan P Lyn RK . MicroRNA-7 mediates cross-talk between metabolic signaling pathways in the liver. Sci Rep (2018) 8:361–1. doi: 10.1038/s41598-017-18529-x PMC5762714 29321595

[34] Zhang W-Z . An association of metabolic syndrome constellation with cellular membrane caveolae. Pathobiol Aging Age Relat Dis (2014) 4:1–8. doi: 10.3402/pba.v4.23866 PMC3926988 24563731

[35] Salle-Teyssières L Auclair M Terro F Nemani M Elsayed SM Elsobky E . Maladaptative autophagy impairs adipose function in congenital generalized lipodystrophy due to cavin-1 deficiency. J Clin Endocrinol Metab (2016) 101:2892–904. doi: 10.1210/jc.2016-1086 27144934

[36] Marat AL Haucke V . Phosphatidylinositol 3-phosphates-at the interface between cell signalling and membrane traffic. EMBO J (2016) 35:561–79. doi: 10.15252/embj.201593564 PMC4801949 26888746

[37] Manna P Jain SK . Phosphatidylinositol-3,4,5-Triphosphate and cellular signaling: Implications for obesity and diabetes. Cell Physiol Biochem (2015) 35:1253–75. doi: 10.1159/000373949 PMC4797942 25721445

[38] Liu P Cheng H Roberts TM Zhao JJ . Targeting the phosphoinositide 3-kinase pathway in cancer. Nat Rev Drug Discovery (2009) 8:627–44. doi: 10.1038/nrd2926 PMC3142564 19644473

[39] Choi S Hedman AC Sayedyahossein S Thapa N Sacks DB Anderson RA . Agonist-stimulated phosphatidylinositol-3,4,5-trisphosphate generation by scaffolded phosphoinositide kinases. Nat Cell Biol (2016) 18:1324–35. doi: 10.1038/ncb3441 PMC5679705 27870828

[40] Waugh C Sinclair L Finlay D Bayascas JR Cantrell D . Phosphoinositide (3,4,5)-triphosphate binding to phosphoinositide-dependent kinase 1 regulates a protein kinase B/Akt signaling threshold that dictates T-cell migration, not proliferation. Mol Cell Biol (2009) 29:5952–62. doi: 10.1128/MCB.00585-09 PMC2772752 19703999

[41] Costello PS Gallagher M Cantrell DA . Sustained and dynamic inositol lipid metabolism inside and outside the immunological synapse. Nat Immunol (2002) 3:1082–9. doi: 10.1038/ni848 12389042

[42] Warshauer JT Lopez X Gordillo R Hicks J Holland WL Anuwe E . Effect of pioglitazone on plasma ceramides in adults with metabolic syndrome. Diabetes Metab Res Rev (2015) 31:734–44. doi: 10.1002/dmrr.2662 25959529

[43] Meikle PJ Summers SA . Sphingolipids and phospholipids in insulin resistance and related metabolic disorders. Nat Rev Endocrinol (2017) 13:79–91. doi: 10.1038/nrendo.2016.169 27767036

[44] Kalderon B Azazmeh N Azulay N Vissler N Valitsky M Bar-Tana J . Suppression of adipose lipolysis by long-chain fatty acid analogs. J Lipid Res (2012) 53:868–78. doi: 10.1194/jlr.M022673 PMC3329386 22338010

[45] Gumieniczek A Owczarek B Pawlikowska B . Oxidative/nitrosative stress and protein damages in aqueous humor of hyperglycemic rabbits: effects of two oral antidiabetics, pioglitazone and repaglinide. Exp Diabetes Res (2012) 2012:653678. doi: 10.1155/2012/653678 22474428 PMC3303562

[46] Sakamaki T Casimiro MC Ju X Quong AA Katiyar S Liu M . Cyclin D1 determines mitochondrial function *in vivo* . Mol Cell Biol (2006) 26:5449–69. doi: 10.1128/MCB.02074-05 PMC1592725 16809779

[47] Zhang Q Sakamoto K Wagner KU . D-type cyclins are important downstream effectors of cytokine signaling that regulate the proliferation of normal and neoplastic mammary epithelial cells. Mol Cell Endocrinol (2014) 382:583–92. doi: 10.1016/j.mce.2013.03.016 PMC3740091 23562856

[48] Song L-Y Ma Y-T Wu C-F Wang C-J Fang W-J Liu S-K . MicroRNA-195 activates hepatic stellate cells *In vitro* by targeting Smad7. BioMed Res Int (2017) 2017:1945631. doi: 10.1155/2017/1945631 28929107 PMC5591989

[49] Kawaguchi K Sakaida I Tsuchiya M Omori K Takami T Okita K . Pioglitazone prevents hepatic steatosis, fibrosis, and enzyme-altered lesions in rat liver cirrhosis induced by a choline-deficient l-amino acid-defined diet. Biochem Biophys Res Commun (2004) 315:187–95. doi: 10.1016/j.bbrc.2004.01.038 15013444

[50] Lian J Fu J . Pioglitazone for NAFLD patients with prediabetes or type 2 diabetes mellitus: A meta-analysis. Front Endocrinol (2021) 12. doi: 10.3389/fendo.2021.615409 PMC8115121 33995271

[51] Ding H Yao J Xie H Wang C Chen J Wei K . MicroRNA-195-5p downregulation inhibits endothelial mesenchymal transition and myocardial fibrosis in diabetic cardiomyopathy by targeting Smad7 and inhibiting transforming growth factor beta 1-Smads-Snail pathway. Front Physiol (2021) 12. doi: 10.3389/fphys.2021.709123 PMC8514870 34658906

[52] Liao H-W Saver JL Wu Y-L Chen T-H Lee M Ovbiagele B . Pioglitazone and cardiovascular outcomes in patients with insulin resistance, pre-diabetes and type 2 diabetes: a systematic review and meta-analysis. BMJ Open (2017) 7:e013927. doi: 10.1136/bmjopen-2016-013927 PMC5223642 28057658

